# 1790. Dethroning Vancomycin: A Multifaceted Stewardship Approach to Reduce Vancomycin in an Academic Medical Center

**DOI:** 10.1093/ofid/ofac492.1420

**Published:** 2022-12-15

**Authors:** Katherine Lusardi, Brett Bailey, Juan Carlos Rico Crescencio, Ramez Awad, Mitchell Jenkins, Ryan K Dare

**Affiliations:** UAMS Medical Center, Little Rock, Arkansas; UAMS, Searcy, Arkansas; University of Arkansas for Medical Sciences, College of Medicine, Little Rock, Arkansas; UAMS Medical Center, Little Rock, Arkansas; University of Arkansas for Medical Sciences, Little Rock, Arkansas; University of Arkansas for Medical Sciences, College of Medicine, Little Rock, Arkansas

## Abstract

**Background:**

Vancomycin is a broadly used antimicrobial, and from 2014-2022 represented the most used antibiotic at our academic medical center. Since 2015, the stewardship program has introduced several measures to optimize vancomycin use including pharmacy consultation, addition of MRSA nares PCR testing, embedding MRSA nares PCR order into vancomycin order panel, and introducing an electronic medical record (EMR) best practice alert to assist providers in identifying patients eligible for vancomycin discontinuation. The impact of each of these interventions are described.

**Methods:**

This is a retrospective, single academic center, comparison of vancomycin utilization over time, following the introduction of various stewardship measures. Each intervention remained in place during subsequent periods. Wilcoxon rank sum was used for analysis.

- Period 1 (P1): 4/2015-12/2016 vancomycin pharmacy consult
- Period 2 (P2): 1/2017-6/2019 pharmacists authorized to order Nasal MRSA PCR
- Period 3 (P3): 7/2019-2/2020 embed nasal MRSA PCR into consult panel
- Period 4 (P4): 2/2020-3/2022 addition of automated EMR time-out

**Results:**

Each period of vancomycin optimization was associated with a statistically significant decrease in vancomycin length of therapy (LOT) in days, when compared to the period before it (P1 vs P2, p< 0.0001; P2 vs P3, p< 0.0001; P3 vs P4, p< 0.0001). Overall institution vancomycin consumption, expressed as monthly days of therapy (DOT)/1000 patient days (PD) also decreased over time, starting with period 3 (P1 vs P2, p=0.4812; P2 vs P3, p=0.0003; P3 vs P4, p=0.0019) (Table 1).

**Table 1:**
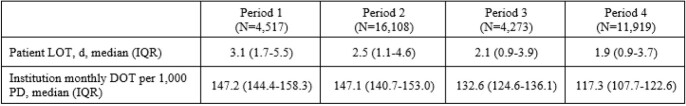
Vancomycin utilization trends over 4 periods of stewardship intervention.

**Conclusion:**

Decreasing vancomycin at an academic medical center is challenging and takes a multifaceted approach with continued efforts over time to maintain a steady downward trend for utilization. These combined efforts have led to vancomycin no longer being the most commonly prescribed antibiotic at our institution.

**Disclosures:**

**All Authors**: No reported disclosures.

